# Optimising Postoperative Pain to Improve Recovery After Lung Cancer Resection: A Narrative Review of Surgical and Analgesic Strategies

**DOI:** 10.7759/cureus.101548

**Published:** 2026-01-14

**Authors:** William Foot

**Affiliations:** 1 Cardiothoracic Surgery, Queensland Health, Brisbane, AUS

**Keywords:** cardiothoracic surgeries, general thoracic surgery, lung cancer surgery, post operative pain management, post thoracotomy pain, regional anesthesiology, video-assisted thoracoscopic surgery (vats)

## Abstract

Surgical resection for lung cancer is the primary treatment modality for early-stage tumours, and there is likely to be an increase in the need for surgery with improvements in the early detection of lung cancer, as well as advances in thoracic surgery that allow patients who would have previously been deemed unsuitable to undergo surgical resection. Despite its potentially curative nature, the invasiveness of thoracic surgery, as well as potential concerns regarding postoperative pain and recovery, means that some patients may decline surgical resection of their lung cancer. Strategies to reduce postoperative pain may improve outcomes following lung resection and may allow more patients to undergo surgical treatment for lung cancer. This narrative review aims to highlight the importance of improving postoperative pain control and review the evidence for some of the surgical and analgesic strategies to achieve this. Modifications to the traditional thoracotomy technique and increased adoption of minimally invasive approaches are some of the surgical strategies that may improve postoperative pain control and recovery. Analgesic strategy may play an even more important role in optimising recovery following thoracic surgery. While opiate medication has been the mainstay of postoperative pain management, there is increasing recognition of opiate-related complications and addiction. Regional anaesthetic techniques are playing a more prominent role in analgesic strategies post-thoracic surgery. Thoracic epidural has been considered the gold standard, but it is associated with its own complications. Newer regional anaesthetic techniques may be equally as effective as a thoracic epidural but with fewer complications. Further study is needed to elicit the optimal regional anaesthesia strategy. Certainly, a combined approach of improvements in surgical technique along with a multi-modal analgesia strategy incorporating regional anaesthetic techniques will yield the best outcomes with regard to reducing postoperative pain, avoiding complications and improving recovery for patients undergoing lung cancer resection.

## Introduction and background

Lung cancer is the leading cause of cancer mortality in the developed world, with five-year survival rates for all lung cancer patients around 25% [1]. Specific lung cancer mortality depends on the stage of lung cancer, with earlier stages having a much more favourable prognosis with treatment compared with advanced-stage lung cancer [2]. Surgery and radiotherapy are the two primary treatment modalities for early-stage lung cancer, and despite efforts to increase the role of radiotherapy as well as other less invasive treatment modalities such as cryo- and microwave ablation for early-stage lung cancer, surgery remains the first-line treatment recommendation for patients with early-stage lung cancer who are deemed operable [3]. Surgery for lung cancer has been shown to have an overall survival advantage over radiotherapy, as well as having the potential to upstage and inform further adjuvant treatments which may provide an additional improved survival advantage over less-invasive treatment modalities such as radiotherapy or ablative treatments which do not yield tissue for pathological assessment of completeness of response to treatment and for further molecular testing [4]. Despite a reduction in smoking rates, the incidence of lung cancer has remained stable over the last decade, indicating an ongoing need for the surgical management of lung cancer [5]. In fact, surgery for the management of lung cancer may increase in the future due to several factors, including increasing early detection of lung cancer as well as expansion of the role of surgery for patients previously not considered for operative management.

The utility of surgery in the treatment of lung cancer depends on the stage of lung cancer at the time of diagnosis. Unfortunately, given the often minimal or non-specific symptoms, lung cancer is often diagnosed late and therefore at an advanced stage where there is a limited role for surgery [6]. Lung cancer screening programmes have been or are being introduced in a number of countries (including Australia) with the goal of increasing the early detection of lung cancer at a stage where surgery as well as other curative treatments can be offered to potentially increase the survival of patients with lung cancer [7]. A recent randomised controlled trial of a lung cancer screening programme showed that, compared with those who were not in a screening programme, participants in a lung cancer screening programme diagnosed with lung cancer were more likely to be at an earlier stage of lung cancer at the time of diagnosis and that their overall and lung cancer-specific mortality were significantly improved [8]. The increase in the detection of early-stage lung cancers will increase the number of patients considered for surgical resection, with the feasibility report for the Australian lung cancer screening programme suggesting that surgeons will see a far greater increase in the number of patients requiring treatment compared with other specialists, such as radiation or medical oncologists involved in the treatment of lung cancer [9].

As well as increasing surgical treatment of lung cancer due to more early detection, there is some suggestion that the utilisation of surgery in the treatment of lung cancer may further increase in the future with expansion into groups previously not considered for surgery, either due to the advanced stage of the cancer at the time of diagnosis, which traditionally ruled them out for surgical management, or due to concerns regarding their ability to tolerate the surgical procedure or the recovery period due to their physical status and comorbidities. Surgery has traditionally been utilised for stage I and II lung cancer with a limited role in stage IIIA lung cancers; however, with increasing sophistication in staging as well as advances in surgical techniques and the increasing use of adjuvant and neoadjuvant therapies, surgery is already playing an increasing role in more advanced lung cancer, and this will likely increase further in the future [10]. Regarding expanding the role of surgery in patients who would previously have been deemed unsuitable for surgery due to concerns regarding their operability, modifications to the surgical techniques to reduce the invasiveness and therefore improve the tolerability of the procedure may allow more patients to undergo surgical resection of their lung cancer. The introduction of minimally invasive techniques as opposed to open techniques has been associated with reduced morbidity and mortality in patients with significant comorbidities who are otherwise borderline operative candidates [11,12]. Lung-sparing techniques may also increase the tolerability of surgery in borderline patients. Lobectomy has been the standard surgical treatment for early-stage lung cancer; however, two landmark studies in recent years have demonstrated the equivalence of sublobar resections and even the superiority of anatomical segmentectomy compared with lobectomy in selected cases with regard to overall survival [13,14]. Given the lesser extent of lung resection involved, this may open the possibility of surgical treatment to patients who would have previously been turned down for lobectomy due to concerns regarding their fitness to tolerate the procedure.

Surgery is certainly more invasive than radiotherapy for the treatment of early-stage lung cancer, and concerns regarding postoperative pain and prolonged recovery can be a significant barrier to a patient’s willingness to undergo surgical treatment of their lung cancer, despite the documented survival advantages over other less invasive treatment modalities. Patient’s perception or expectation of a reduced quality of life following surgery has been shown to correlate with their refusal to undergo surgical management of otherwise operable early-stage lung cancer, which may affect their long-term survival [15]. Strategies to improve recovery and pain following lung resection may alleviate these concerns and encourage patients to undergo recommended surgical treatment and improve overall survival in this group.

## Review

Literature search and review strategy

A comprehensive literature search was performed to identify studies evaluating postoperative pain following lung cancer resection with an emphasis on the consequences of postoperative pain and the surgical and analgesic strategies to improve pain control. Electronic databases queried included PubMed, Embase, and Google Scholar. The key words used in the search strategy included thoracic surgery, lung cancer surgery, thoracotomy, video-assisted thoracic/thoracoscopic surgery (VATS), pain, postoperative pain, chronic pain, enhanced recovery after surgery (ERAS), analgesia, multi-modal analgesia, regional analgesia and blocks (including the specific terms 'thoracic epidural', 'paravertebral', 'erector spinae', 'serratus anterior' and 'intercostal nerve blocks'). Reference lists from identified papers were manually searched to identify further relevant studies. Additional studies were identified from relevant international society guidelines and publications. Results were limited to English language studies with an emphasis on clinical studies, including randomised controlled trials, observational and retrospective studies, reviews and meta-analyses, and guideline documents. Case reports, conference abstracts, and non-human studies were excluded, as well as studies involving experimental or novel treatments that are not already in clinical use, to limit the results to current clinical practices. Due to the broad topic and significant heterogeneity in study design, definitions, and outcome measures, surgical techniques described, analgesic regimens, and confounders in the identified studies, results were synthesised as a narrative review and grouped thematically as consequences of postoperative pain and surgical and analgesic strategies to reduce postoperative pain.

Consequences of poorly controlled postoperative pain

Thoracic surgery is considered one of the most painful of all surgical procedures [16]. The chest wall and thoracic cavity have dense sensory innervation, and the mechanism for increased pain following thoracic surgery is thought to be due to incision of the chest wall muscles, retraction/division of the ribs, injury to the intercostal nerve, pleural irritation, and the placement of chest drains [17]. Uncontrolled pain following thoracic surgery has been associated with increased respiratory complications, prolonged hospital length of stay, reduced quality of life, and subsequent chronic pain [18]. The development of chronic pain syndromes (post-thoracotomy pain syndrome [PTPS]) has been reported to occur in between 25% and 60% of patients undergoing thoracic surgery [19]. PTPS is generally defined as "pain at the thoracotomy site persisting after two months", which is open to significant interpretation, and the true incidence of permanent or longstanding significant chronic pain syndromes following thoracic surgery is probably lower than these quoted figures given that on longer-term follow-up there is progressively less reported ongoing pain over time in patients initially diagnosed with PTPS [20]. Despite this, a thoracotomy is still amongst the highest-risk procedures for the development of chronic pain [21]. A number of risk factors exist for the development of PTPS, including age, gender, pre-existing pain syndromes, psychosocial factors, recurrence, and need for further treatment [19]. The level of acute pain experienced during and following the index operation is considered one of the more influential modifiable risk factors for the development of PTPS, and considerable interest has been shown in developing ways to reduce the levels of acute pain post thoracic surgery through either adjustments to the surgical technique or to the perioperative analgesia regimen in order to improve the short-term outcomes following lung resection as well as avoid the development of chronic pain syndromes [22]. This is especially important as there is growing recognition of the importance of quality of life (QOL) in cancer survivorship, with chronic pain issues ranking highly as a significant issue for survivors of cancer treatment [23]. Given that PTPS is associated with significantly worse QOL than those who did not suffer chronic pain post their lung cancer surgery, it highlights the need to reduce the incidence of PTPS [24].

Surgical strategies to improve postoperative pain and recovery

Various modifications to the technique of thoracotomy have been proposed to reduce the trauma and, therefore, the pain of the incision. Muscle-sparing thoracotomy (whereby division of the latissimus dorsi and serratus anterior muscles is avoided) was initially thought to produce less pain compared with a muscle-splitting thoracotomy; however, while there is reasonable evidence for improved shoulder function with the use of this incision, evidence for reduced pain is mixed [25,26]. Injury to the intercostal nerve from the thoracotomy incision, either by the use of rib-spreading or during closure of the thoracotomy wound, has been implicated in both the level of acute pain as well as the development of chronic pain syndromes [19]. Modified thoracotomy techniques that reduce this injury, such as harvesting an intercostal flap before rib-spreading and suture techniques for reapproximating the rib space that avoid compression of the intercostal nerve by either the retractor or the pericostal closure sutures, have been shown to significantly reduce the level of postoperative pain in several randomised controlled trials when compared with standard thoracotomy techniques; however, even with these techniques, pain scores following thoracotomy are still significant in the immediate postoperative period [27-29].

Video-assisted thoracic surgery (VATS) was introduced as a minimally invasive technique for lung resection as a means of avoiding thoracotomy and its associated complications, which gained increasing popularity from the 1990s onwards [30]. Proponents argued that the advantages of VATS compared with open surgery for lung cancer included fewer complications, less pain, and therefore a faster recovery [31]. However, its initial acceptance as a feasible technique for anatomical lung resection was limited owing to concerns regarding the safety of the procedure, the ability to perform a complete oncological operation, and the cost when compared with a lung resection by thoracotomy [32]. Support for VATS was further limited with an early randomised trial demonstrating no significant benefits over thoracotomy [33]. Despite this, interest in VATS continued to grow, with more surgeons performing minimally invasive lung resections, although reported results were mixed. Proponents of VATS argued that non-standardisation of the technique, with multiple variations in how the surgeries were performed with regard to the number of ports, size of incisions, and the use of rib spreading, amongst other factors, influenced the outcomes of VATS when compared with thoracotomy [34]. The CALGB 39802 trial was the first to evaluate a standardised technique for VATS resection of lung cancer, specifying one 4-8 cm utility incision, a further two 0.5-1 cm ports, and the avoidance of rib spreading [35]. This trial confirmed the feasibility of VATS as an acceptable technique for lung cancer resection with similar outcomes. Following this, the proportion of lung cancer resections performed via the VATS approach has increased over time [36]. Associated with this has been the publication of an increasing number of studies highlighting the potential benefits of VATS over thoracotomy, including reduced inflammatory response and pain, reduced duration of chest tube drainage and prolonged air leak, reduced respiratory and overall complications and at least equivalent if not improved survival and oncological outcomes, although these have been criticised as being largely single-centre retrospective reports or small, low-quality randomised trials [37]. To address this, two major randomised controlled trials have been conducted to compare VATS vs thoracotomy for lung resection, particularly with regard to pain and recovery. The first of these was a study from Denmark comparing four-port VATS with anterolateral thoracotomy, specifically looking at pain and QOL [38]. It showed that compared with the thoracotomy group, patients who underwent VATS lung cancer resections had significantly reduced pain, although this was most pronounced early on and equalised four weeks post-surgery. Furthermore, there was no difference between the groups when comparing the number of patients who specifically reported severe pain levels. With regard to quality-of-life measures, the study showed mixed results, with significant differences in favour of VATS noted with one type of QOL measure but not with another. Safety and oncological outcomes were equivalent between techniques. The second larger randomised controlled trial is the VIOLET trial from the UK comparing posterolateral thoracotomy with VATS (1-4 ports) [39]. Interestingly, participants were blinded to their treatment allocation during their hospital stay and were asked to guess their treatment allocation prior to and on discharge, before being informed. The primary outcome measure of physical functioning at five weeks showed a statistically significant benefit to VATS, but this did not meet the pre-specified clinical threshold. With regard to pain scores, there was no difference between VATS and thoracotomy on day one following surgery; however, from day two onwards, there was a significant improvement in pain scores with VATS. When pain scores were adjusted for analgesia requirements, there was a significant benefit for VATS. When looking at the longer-term trends, there seemed to be an early advantage towards VATS with reduced pain scores and improved QOL metrics, with thoracotomy catching up between five weeks and three months for most metrics, suggesting a more rapid recovery with VATS compared with thoracotomy. Interestingly, with regard to the participants' perception of what procedure they underwent, less than half of all the participants at both two days post-procedure and on discharge correctly guessed their treatment assignment. In fact, fewer of the thoracotomy group correctly guessed their assignment between thoracotomy and VATS compared with the VATS group, suggesting that most of the thoracotomy group believed they had undergone a minimally invasive procedure as opposed to the open operation they received. This might suggest that there isn’t as significant a perceptible difference to the patient in the immediate postoperative period between the two techniques when the patient is unaware whether they have undergone an open or minimally invasive procedure, and that the patients’ expectations of what level of pain they are going to experience may influence their perceived pain and recovery. With regard to safety outcomes, there were significantly fewer overall adverse events with VATS, although no difference in severe adverse events. Oncological outcomes showed no difference in overall survival, progression-free survival, or time to uptake of eligible adjuvant therapy. Overall, this trial confirms the safety of VATS for lung cancer resection with the benefit of improved early postoperative recovery over thoracotomy, although the short-term and somewhat modest benefits shown have led some authors to conclude that these papers confirm the equivalency of VATS to open lobectomy but fail to demonstrate any clear superiority [40]. With regard to minimising long-term pain issues, a prospective study looking at the incidence of chronic pain following lung resections by either open thoracotomy or VATS demonstrated no difference in the development of or the severity of PTPS up to six months [41]. Regardless of proven benefits, patients increasingly want minimally invasive surgical treatment of their lung cancer and would consider other forms of treatment, such as radiotherapy over open surgery, if VATS were unavailable [42]. Along with patient desire, VATS approaches are increasingly being recommended as the preferred surgical approach by many society guidelines and, as a consequence of these factors, are increasingly becoming the preferred approach by thoracic surgeons for lung cancer surgery [43]. Further advances in minimally invasive thoracic surgery, such as uniportal VATS or robotic techniques, may further improve the pain and recovery following lung resection and increase the benefits of minimally invasive surgery over traditional techniques for patients with lung cancer [44] (Table 1).

**Table 1:** Summary of evidence for surgical techniques to reduce postoperative pain

Comparison	Key outcome	Study design	Findings	Comments	Key references
Modified thoracotomy techniques					
Muscle-sparing vs. muscle-splitting thoracotomy	Acute postoperative pain	Observational studies, small RCTs	Inconsistent effect on pain, improved shoulder function	Heterogeneous techniques, inconsistent results	[25,26]
Modified thoracotomy (nerve-sparing) vs. standard thoracotomy	Acute postoperative pain	Multiple RCTs	Reduced early postoperative pain	Consistent benefit, nil blinding, residual pain remains	[27–29]
Video assisted thoracic surgery (VATS)					
VATS vs. thoracotomy (General evidence)	Early recovery and complications	Systematic reviews, retrospective studies, small RCTs	Improved early recovery with VATS	Predominantly retrospective data, potential selection and centre bias	[36,37]
VATS (four-port) vs. anterolateral thoracotomy (Danish trial)	Acute postoperative pain	RCT	Reduced early pain, no difference at 4 weeks	Pain benefit time-limited, QOL results inconsistent	[38]
VATS vs. posterolateral thoracotomy (VIOLET trial)	Pain and physical function	Large multicentre RCT with blinding	Pain same day 1, improved from day 2, modest functional benefit	Large study, well blinded, patients could not identify which arm they were allocated to	[39]
VATS vs. thoracotomy	Chronic post-thoracotomy pain (PTPS)	Prospective cohort study	No difference at 6 months	Non-randomised	[41]
Emerging techniques					
Uniportal VATS, robotic)	Acute pain and recovery	Early observational and comparative studies	Potential benefit	Early-phase data, lack of RCTs, selection bias, learning-curve effects	[44]

Analgesic strategies to improve postoperative pain and recovery

Along with advances in surgical techniques, there has been increasing interest in multimodality enhanced recovery after surgery (ERAS) protocols, which have been shown to be effective in reducing complications, treatment costs, and length of stay following thoracic surgery [45]. These protocols involve multiple multidisciplinary interventions across the patient’s surgical path from referral to perioperative to discharge, which have a synergistic effect greater than their sum parts [46]. Optimisation of a patient’s analgesia is an integral part of any ERAS protocol, and recent studies have shown that with an aggressive enhanced recovery package following thoracic surgery, equivalent outcomes for VATS and open lobectomy could be achieved in both the short and longer term with regard to postoperative pain scores, analgesic requirements, and length of stay [47,48]. This may suggest that analgesic strategy rather than operative modality may have a greater role in optimising a patient's recovery following lung resection.

Given the levels of pain associated with thoracic surgery, effective analgesia is a critical component of the care of patients undergoing lung cancer resection, with ineffective pain relief associated with increased respiratory complications, ICU admissions and length of stay as well as the development of chronic pain syndromes [49]. Opiate analgesia has traditionally formed the basis of postoperative pain relief regimes; however, there has been increasing awareness of the influence of opiates on respiratory complications through their respiratory depressant effects, as well as a growing link between opiate use postoperatively and the development of long-term dependence and addiction issues [50]. Multi-modal analgesia regimes with the addition of non-opiate analgesic medications as well as other adjuncts such as regional anaesthesia are effective methods of achieving opiate-sparing or opiate-free analgesia. Paracetamol is a commonly used analgesic which can reduce opiate consumption by 20% with minimal adverse effects and is generally indicated in all post-surgical patients [51]. Non-steroidal anti-inflammatory drugs (NSAIDs) have also been shown to be effective in reducing post-thoracotomy pain and, when used in combination with paracetamol, have an overall analgesic effect greater than that of each drug individually [52,53]. Adverse effects of NSAIDs, including increased bleeding and renal impairment, have resulted in some caution with regard to their routine use in postoperative patients [54]. The use of neuropathic analgesic agents such as gabapentin and pregabalin, as well as the use of sedative agents such as ketamine and dexmedetomidine, has had mixed results in small studies, with the level of benefit insufficient for their recommendation for routine use in postoperative analgesia regimens [55].

Regional anaesthesia forms a major component of any multi-modal analgesia strategy where local anaesthetic is delivered either to the operative area or to the area of sensory innervation of the operative site to induce analgesia through blockade of pain sensation. In thoracic surgery, this can be achieved either by thoracic epidural or increasingly via regional plane blocks, of which the most commonly used include paravertebral, erector spinae, serratus anterior and intercostal nerve blocks [56]. Regional anaesthesia as part of a multi-modal analgesia strategy is strongly recommended in ERAS protocols for thoracic surgery, and while a thoracic epidural was defined in early ERAS protocols as the necessary regional anaesthesia technique of choice, the latest protocol strongly recommends the use of regional anaesthesia without specifying the technique by which this is achieved [57]. A thoracic epidural, where local anaesthetic is injected directly into the epidural space to act directly on the nerve roots either continuously or in a patient-controlled manner, is one of the oldest regional anaesthetic techniques in thoracic surgery and has been considered the gold standard until recently. Its use in thoracic surgery has been shown to reduce pain score, opiate consumption, hospital length of stay and complications in both open thoracotomy and VATS procedures in multiple studies, although this has not been universal [49]. Greater awareness of the adverse effects of epidural anaesthesia, including urinary retention, lower limb weakness, and hypotension, as well as the restriction on mobilisation by the nature of patient attachment to the delivery device, has resulted in increased interest in other regional anaesthetic techniques [58]. The main other regional anaesthetic techniques used in thoracic surgery include the paravertebral, erector spinae, serratus anterior and intercostal nerve blocks, which also have the advantage that they can be used in patients who have contraindications to epidural placement. Each of these techniques has studies confirming their efficacy when compared with no regional anaesthesia [59-62]. Paravertebral blocks (PVB) have been shown to have an equivalent analgesic effect to thoracic epidurals in several studies with fewer minor and major side effects and no difference in postoperative complications or overall mortality [63,64]. Erector spinae blocks (ESB) work by a similar mechanism to PVB but with less risk of pneumothorax due to the different anatomical infiltration site and have been shown in some studies to have a similar effect to both thoracic epidural and PVB [65,66]. This effect is not universally reported, with other studies showing inferior analgesia with ESB compared with PVB in patients undergoing VATS [67]. Serratus anterior blocks have a more limited area of effect than other blocks and generally have been shown to be less effective than thoracic epidurals as well as both PVB and ESB, but may have a role in uniportal VATS [68,69]. All of these techniques can either be administered as a single shot or continuous infusion and can either include local anaesthetic alone or with additives (such as opiates or dexmedetomidine), and studies comparing these have been mixed, leading to no strong recommendation either way with regards to these variations, although there are concerns with the use of continuous regional anaesthetic techniques limiting patient mobilisation [55]. Intercostal nerve blocks (ICNB), either performed percutaneously by the anaesthetist or more commonly by the surgeon directly, are very simple to perform and have been shown not to add significantly to the operative time, which is in contrast to the other forms of regional anaesthesia, which require additional skills and training for the anaesthetist and can add significantly to the anaesthetic portion of the operation time [70]. They have been shown to be superior to parenteral analgesia alone and have been found to have similar efficacy to more complex forms of regional anaesthesia in several studies [71]. While some smaller studies have shown that ICNBs have less efficacy compared with TEA or PVBs and, as such, have a lower level of recommendation than other regional anaesthesia techniques, a larger trial concluded that ICNB was not inferior to TEA in thoracic surgery [72]. Cryoanalgesia is not recommended in the latest ERAS protocol, as it has been implicated in the development of chronic pain, although there has been renewed interest in this area recently [57,73]. The main limitation of all of these studies comparing different anaesthetic techniques is the confounding factor of different surgical techniques/postoperative routines and their impact on postoperative pain. In these studies, not only were different regional anaesthetic techniques compared in either VATS or thoracotomy, making it difficult to compare between studies, but also differences in individual surgeons’ methods of performing the same operation as well as differences in postoperative care make it difficult to tease out the specific impact of the different regional anaesthetic techniques from the influence of differences in surgical procedure and postoperative management. Future studies should consider standardising or at least controlling for differences in surgical techniques and postoperative management strategies to truly compare the effectiveness of different regional anaesthetic techniques in thoracic surgery (Table 2).

**Table 2:** Summary of evidence for analgesic techniques to reduce postoperative pain

Intervention	Study design	Findings	Comments	Key references
Enhanced Recovery Pathway				
ERAS protocols with multimodal analgesia	Prospective and retrospective studies	Reduced complications, length of stay, opiate use, improved recovery	Synergistic effect of multiple interventions, difficult to isolate analgesic contribution	[45–48]
Oral Analgesics				
Paracetamol	RCTs / clinical studies	Reduces opioid consumption by ~20%, minimal adverse effects	Generally safe and widely recommended	[51]
NSAIDs ± paracetamol	RCTs / clinical studies	Effective analgesia, additive to paracetamol	Risk of bleeding and renal impairment	[52–54]
Other agents (gabapentin, pregabalin, ketamine, dexmedetomidine)	Small RCTs / observational	Mixed results, limited evidence	Insufficient evidence for routine use	[55]
Opiates	Clinical studies / RCTs / Reviews	Effective analgesia	Side effects and dependence issues	[50]
Regional anaesthetic techniques				
Thoracic epidural analgesia	Multiple RCTs, observational	Reduces pain, opioid use, LOS, complications	Gold standard, adverse effects include hypotension, urinary retention, limited mobility	[49,58]
Paravertebral block	RCTs / cohort studies	Equivalent analgesia to TEA, fewer side effects	Effective alternative to TEA, good safety profile	[63,64]
Erector spinae block	RCTs / observational	Comparable to PVB / TEA in some studies; less pneumothorax risk	Mixed efficacy, some studies show inferior analgesia in VATS	[65–67]
Serratus anterior block	Observational / small RCTs	Limited analgesic coverage, less effective than TEA/PVB/ESB	Potential role in uniportal VATS	[68,69]
Intercostal nerve block	RCTs / prospective studies	Comparable analgesia to TEA/PVB in some studies, simple, rapid	Lower level recommendation historically, variable results	[70–72]

## Conclusions

Surgery plays a key role in the management of early-stage lung cancer, and due to several factors, the number of patients requiring lung cancer resection is likely to increase in the future. Poor postoperative pain control is associated with increased complications, worse outcomes, and reduced quality of life and is unacceptable in the current era. Surgical strategies, including modifications to surgical technique and a transition to minimally invasive surgery, improve pain control and quality of life as well as reduce complications. Analgesic strategies, including enhanced recovery protocols and multi-modal analgesia incorporating regional anaesthetic techniques, dramatically improve pain control, reduce postoperative complications, perhaps to an even greater extent than surgical technique, and should be a mandatory component of modern thoracic surgical practice. Ongoing collaboration between thoracic surgery and anaesthetic disciplines, as well as the wider multidisciplinary team, is critical to the ongoing refinement of both components to truly optimise pain control and recovery following lung cancer resection surgery to the patients' benefit.
